# Clinical Trial: Predicting Response to Iron Therapy in Patients With Active Inflammatory Bowel Disease Using Hepcidin and Functional Iron Indices: A Multicentre Randomised Trial

**DOI:** 10.1111/apt.70775

**Published:** 2026-06-08

**Authors:** Lola J. M. Koppelman, Roberta Loveikyte, Rogier L. Goetgebuer, Sander van der Marel, Annemarie C. de Vries, Gerard Dijkstra, Andrea E. van der Meulen‐de Jong, the Dutch Iron Study Group, M. Duijvestein, M. van der Have, C. Horjus, W. G. N. Mares, Z. Mujagic, B. Oldenburg, R. West

**Affiliations:** ^1^ Department of Gastroenterology and Hepatology Leiden University Medical Center Leiden the Netherlands; ^2^ Department of Gastroenterology and Hepatology Amsterdam University Medical Center Amsterdam the Netherlands; ^3^ Department of Gastroenterology and Hepatology Haaglanden Medical Center Den Haag the Netherlands; ^4^ Department of Gastroenterology and Hepatology Erasmus University Medical Center Rotterdam the Netherlands; ^5^ Department of Gastroenterology and Hepatology University Medical Center Groningen, University of Groningen Groningen the Netherlands

**Keywords:** anaemia, hepcidin, inflammatory bowel disease, iron deficiency, iron therapy, personalised medicine

## Abstract

**Background:**

Iron deficiency anaemia (IDA) is common in inflammatory bowel disease (IBD) and impairs quality of life. Hepcidin, the regulator of systemic iron homeostasis, may predict response to iron therapy; however, its utility in IBD remains unclear. This study evaluated whether baseline hepcidin predicts response to oral and intravenous (IV) iron in active IBD to guide personalised treatment.

**Methods:**

Ninety adults with active IBD and iron deficiency (with or without anaemia) from two randomised trials received IV iron, oral ferrous fumarate (FF) or oral ferric maltol (FM). Response at 12 weeks was defined as haemoglobin increase ≥ 1.2 mmol/L (19.3 g/L) or normalisation in IDA or ferritin > 100 μg/L and transferrin saturation > 20% in iron deficient patients. Baseline iron indices including ferritin, hepcidin, and soluble transferrin receptor (sTfR) were measured. Logistic regression and receiver operating curve analyses evaluated predictive performance.

**Results:**

Baseline hepcidin strongly predicted response to iron therapy: AUC_(FF)_: 0.86, 95% CI: 0.71–1.00 and AUC_(IV)_: 0.63, 95% CI: 0.30–0.96. Hepcidin > 2.68 μg/mL identified non‐responders to FF with 89% sensitivity and 77% specificity. Each twofold increase in baseline hepcidin or ferritin reduced the odds of response [log_2_(hepcidin) OR: 0.71, 95% CI: 0.56–0.89; log_2_(ferritin) OR: 0.38, 95% CI: 0.21–0.69]. Higher transferrin/log_10_(ferritin) (OR: 2.95, 95% CI: 1.38–6.30) and sTfR/log_10_(ferritin) ratios (OR: 1.29, 95% CI: 1.05–1.59) increased likelihood of response.

**Conclusion:**

Baseline hepcidin supports route selection for iron therapy: higher levels favour IV iron, while lower levels indicate likely oral response. Ferritin‐based indices, notably transferrin/log_10_(ferritin), offer pragmatic alternatives where hepcidin testing is unavailable.

**Trial Registration:**

ClinicalTrials.gov identifier: NCT05581420 and NCT05456932

## Graphical abstract

Hepcidin levels predict response to iron therapy and support route selection for iron therapy in patients with active inflammatory bowel disease: higher levels favour IV iron, while lower levels indicate likely oral response. Ferritin‐based indices, particularly transferrin/log_10_(ferritin) and sTfR/log_10_(ferritin), offer reliable alternatives.

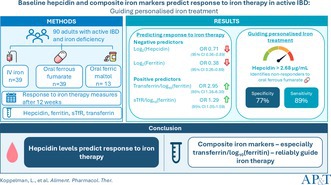

## 1 Introduction

Anaemia is one of the most frequent extraintestinal complications of inflammatory bowel disease (IBD), encompassing both ulcerative colitis (UC) and Crohn's disease (CD) [1]. These relapsing, immune‐mediated disorders are characterised by chronic gastrointestinal inflammation that can impair nutrient uptake, increase nutrient demands and drive systemic metabolic changes [2, 3]. The estimated prevalence of anaemia in IBD varies, ranging from 9% to 74% depending on the patient subpopulation [4]. A recent evaluation of anaemia prevalence among Dutch patients showed that 18% of outpatients were anaemic and half of the patients included in the study had iron deficiency (ID) [5]. Anaemia is associated with reduced quality of life, diminished cognitive and physical performance, impaired work participation and greater healthcare utilisation [6, 7].

Iron plays a central role in erythropoiesis, oxygen transport, oxidative metabolism and mitochondrial energy production; therefore, ID and iron deficiency anaemia (IDA) are substantial contributors to fatigue and functional impairment. Reflecting this burden, IBD guidelines recommend routine screening and early anaemia treatment with the goal of fully restoring iron stores [8].

Both oral and intravenous (IV) iron therapies are effective, yet each has limitations. IV formulations reliably improve haemoglobin and ferritin but require day‐case admission and have been associated with hypophosphatemia and infrequent serious adverse events [9, 10, 11]. Traditional oral iron salts are inexpensive and widely accessible but can cause gastrointestinal adverse effects, limiting adherence [12]. Moreover, oral iron absorption might be hindered by the state of inflammation.

Hepcidin, the regulator of systemic iron homeostasis, has gained attention as a potential biomarker to guide iron therapy in IBD [13, 14, 15]. By controlling the expression of ferroportin, hepcidin regulates the degree of intestinal iron absorption and iron mobilisation from the reticulo‐endothelial system and hepatic stores [16]. Hepcidin expression increases in response to inflammation or iron excess, leading to iron being retained within storage sites, reducing intestinal iron absorption and decreasing its availability for erythropoiesis, whereas ID and enhanced erythropoietic activity suppress production of hepcidin. ID and inflammation often co‐exist in patients with IBD, leading to a dilemma regarding iron status optimalisation. Studies indicate that, despite active inflammation, an iron‐deficient state is the dominant driver of hepcidin levels [14, 17]. Studies in different patient populations suggest that hepcidin may predict response to both oral and IV iron, yet this relationship has not been explored in active IBD [18, 19]. In addition to hepcidin, composite iron markers such as the transferrin/log_10_(ferritin) and sTfR/log_10_(ferritin) ratios have been proposed as useful markers for the diagnosis of IDA [20, 21]. By integrating measures of iron demand and storage, these indices may better reflect functional iron availability. Indirectly they may capture hepcidin‐driven iron restriction, thereby potentially aiding in the prediction of response to iron therapy.

This study investigates whether hepcidin and composite iron markers can serve as a predictor of therapeutic response to oral and IV iron in patients with active IBD receiving immunosuppressive treatment. By examining iron status, inflammation and treatment outcomes, this study tries to better understand the role of hepcidin in the management of ID(A) in IBD in order to personalise and optimise iron therapy.

## 2 Materials and Methods

### 2.1 Study Design and Participants

Data from two multicentre, open‐label, randomised controlled trials, OVI‐IBD (ClinicalTrials.gov identifier: NCT05581420) and PRIme (ClinicalTrials.gov identifier: NCT05456932) [22], were pooled for this analysis. Both studies enrolled adults (≥ 18 years) with active IBD and either IDA (OVI‐IBD and PRIme) or ID (PRIme) across multiple general, teaching and academic hospitals in the Netherlands. Active IBD was defined by clinical, endoscopic or biochemical activity, including C‐reactive protein (CRP) > 5 mg/L or faecal calprotectin (FCP) > 150 mg/kg. Key exclusion criteria were recent blood transfusion, contraindications to iron supplementation and significant comorbidities affecting iron metabolism, as detailed in the Prime protocol [22]. All participants provided written informed consent prior to inclusion. Both trials were approved by the appropriate medical ethics review committee and were conducted in accordance with the principles of the Declaration of Helsinki. Patients included in the current analysis were those enrolled between 2021 and July 2024.

### 2.2 Randomisation and Interventions

Participants were randomly assigned to one of three iron supplementation strategies: intravenous iron suppletion (IV), oral ferrous fumarate (FF) or oral ferric maltol (FM). In the PRIme study, participants were randomised (1:1:1) to FM, IV or FF groups [22], while in OVI‐IBD, participants were randomised (1:1) to IV or FF groups. Randomisation was stratified by study centre (i.e., academic or not), by disease activity (moderate disease activity defined as either CRP < 20 mg/L or FCP < 300 mg/kg versus severe disease activity defined as either CRP > 20 mg/L or FCP > 300 mg/kg), and by expected induction therapy (i.e., no induction therapy, expected dose increase in prednisone (derivates) during the study period, and expected dose increase of biologicals/small molecules during the study period).

FM was administered as 30 mg twice daily for 12 weeks. In the OVI‐IBD trial, FF was given as 200 mg daily with dose halving after haemoglobin normalisation at weeks 4 or 12, in accordance with Dutch clinical guidelines. FF dosing was fixed in the PRIme trial [23]. IV iron was administered according to local practice, using available formulations and dosed per the Dutch national formulary [24, 25]. Dosing was based on haemoglobin level and body weight: patients with haemoglobin < 6.2 mmol/L (99.9 g/L) received 1500 mg (35–70 kg) or 2000 mg (≥ 70 kg), and those with haemoglobin 6.2–8.7 mmol/L (99.9–140.2 g/L) received 1000 mg (35–70 kg) or 1500 mg (≥ 70 kg).

### 2.3 Outcomes

The primary outcome was the predictive value of baseline hepcidin levels for treatment response to iron therapy at 12 weeks. Treatment response in patients with IDA was defined as either an ≥ 1.2 mmol/L increase in haemoglobin or haemoglobin normalisation (≥ 7.3 or ≥ 8.0 mmol/L (117.6 or 128.9 g/L) depending on sex); in patients with ID at baseline, the response was defined as ferritin > 100 μg/L and transferrin saturation (TSAT) > 20%.

Secondary outcomes included early treatment response at week 5, normalisation of iron stores at 12 weeks (ferritin > 100 μg/L and TSAT > 20%), and changes in iron‐related biomarkers over time. Alternative response predictors, such as ferritin or transferrin/log_10_(ferritin) ratio, were explored. Treatment adherence to oral iron was assessed and compared between responders and non‐responders with a monthly questionnaire (MMAS‐8) [26, 27, 28].

### 2.4 Data Collection and Monitoring

Laboratory assessments were conducted at baseline, at week 4 (OVI‐IBD) or week 6 (PRIme) and at week 12. The week 4 and 6 measurements were pooled and analysed as a single intermediate time point (week 5). The following biochemical parameters were measured: haemoglobin, ferritin, serum iron, transferrin, total iron‐binding capacity (TIBC), TSAT, hepcidin, soluble transferrin receptor (sTfR), zinc protoporphyrin (ZPP), erythropoietin (EPO), CRP, white blood cell count (WBC), platelet count, interleukin‐6 (IL‐6), reduced sulfhydryl groups (R‐SH) and FCP. Moreover, transferrin/log_10_(ferritin) ratio and the sTfR/log_10_(ferritin) ratio were calculated given the previously reported diagnostic value [20, 21]. All (serious) adverse events ((S)AEs) were documented and evaluated for their relation to the intervention. All data were monitored by an independent study monitor and managed using a secure electronic data capture system.

### 2.5 Serum Sample Analysis

Routine biochemical parameters were measured in the hospital's accredited clinical laboratory. Hepcidin, sTfR, ZPP, IL‐6, EPO and R‐SH were not part of routine testing and were quantified in serum samples collected at baseline and week 12. Samples from the PRIme trial were stored directly at −80°C, whereas samples from the OVI‐IBD trial were initially stored short‐term at −20°C before transfer to −80°C.

Hepcidin was measured using an enzyme‐linked immunosorbent assay (ELISA; R&D Systems/Bio‐Techne, DPH250). sTfR was quantified using an ELISA kit (BioVendor, RD194011100); it reflects cellular iron demand and erythropoietic activity [21]. ZPP, a marker of impaired iron incorporation into haem and functional ID [29], was measured using an ELISA kit (MyBioSource, MBS167672). IL‐6, a key pro‐inflammatory cytokine and major inducer of hepcidin expression [30], was quantified using a high‐sensitivity ELISA (R&D Systems/Bio‐Techne, HS600C). EPO, reflecting erythropoietic drive and upregulated in response to hypoxia and anaemia [31], was measured using an ELISA kit (Abbexa, ABX151450). Systemic oxidative stress was assessed by measuring circulating free thiols (R‐SH), which decrease in the presence of increased reactive oxygen species, using a previously described colorimetric method [32].

All assays were performed according to the manufacturer's instructions after optimisation of sample dilutions.

### 2.6 Statistical Analysis

Descriptive statistics were used to summarise baseline characteristics across treatment groups. Categorical variables were reported as counts and percentages, while continuous variables were reported as medians with interquartile ranges (IQR) or means with standard deviations (SD), as appropriate. For comparisons across treatment groups, one‐way ANOVA was used for normally distributed continuous variables. For non‐normally distributed data, the Kruskal–Wallis test was applied. Categorical variables were compared using the χ^2^ test. The Benjamini‐Hochberg procedure was used to adjust for multiple testing, considering significance under a false discovery rate of 5%. Treatment response rates at weeks 5 and 12 were calculated per group. Paired and Wilcoxon signed‐rank tests were applied, as appropriate, to assess differences in biochemical changes over time between responders and non‐responders to iron therapy.

Logistic regression analyses were used to evaluate the association of baseline biomarkers in relation to iron therapy, with odds ratios (ORs) and 95% confidence intervals (CIs) reported. Receiver operating characteristic (ROC) curve analyses were performed to evaluate the discriminative ability of baseline biomarkers for predicting treatment response, both overall and stratified by treatment group. Area under the curve (AUC), optimal thresholds, sensitivity and specificity were reported for each marker.

All statistical analyses and data visualisation were performed using R (version 2025.05.0 + 496), with a two‐sided *p*‐value < 0.05 considered statistically significant [33].

## 3 Results

### 3.1 Baseline Characteristics

Ninety patients were enrolled in the study: 39 received IV iron, 38 received FF, and 13 FM. During the study period, 15 participants were lost to follow‐up or withdrew their consent after randomisation; last available data was included for all participants in the statistical analyses. Baseline characteristics are shown in Table 1. The overall median age was 40 years (IQR: 30.0–56.8), and 58.9% of participants were female. Approximately 58% of the cohort were diagnosed with CD. All 90 participants had mild to moderate disease activity, with a median FCP 521 μg/g (IQR: 193–1272). There were no significant differences between the IV and FF groups, except for patients in the FM group, who received monotherapy with aminosalicylates more often compared with patients in the other groups. Missing data at baseline, week 5 and 12 is reported in Table S2.

**TABLE 1:** Baseline characteristics.

	Total (*n* = 90)	IV iron (*n* = 39)	Ferrous fumarate (*n* = 38)	Ferric maltol (*n* = 13)	*p*
Age (years)	40.0 [30.0–56.8]	40.0 [26.5–57.5]	44.5 [30.3–59.3]	38.0 [24.0–42.0]	0.411
Female sex	53 (58.9%)	22 (56.4%)	22 (57.9%)	9 (69.2%)	0.709
Disease duration (years) (Median [Q1–Q3])	10.0 [3.0–17.8]	9.0 [3.5–17.5]	10.5 [3.0–18.3]	12.0 [8.0–15.0]	0.785
IBD type (CD)	55 (57.8%)	26 (66.7%)	22 (57.9%)	4 (30.8%)	0.076
Medication use					
No medication	3 (3.3%)	2 (5.1%)	0 (0.0%)	1 (8.33%)	0.291
Corticosteroids^a^	8 (8.9%)	4 (10.3%)	3 (7.9%)	1 (7.7%)	0.933
Aminosalicylates^a^	7 (7.8%)	2 (5.3%)	2 (5.3%)	3 (23.1%)	0.012
Thiopurines/MTX^a^	2 (2.2%)	1 (2.6%)	1 (2.6%)	0 (0.0%)	0.839
Biologicals/Small molecules	75 (8.3%)	33 (84.6%)	33 (8.7%)	8 (61.5%)	0.069
History of surgery	26 (28.9%)	13 (33.3%)	10 (26.3%)	3 (23.1%)	0.701
(Partial) colectomy	7 (7.8%)	2 (5.1%)	4 (10.5%)	1 (7.7%)	0.765
Ileocecal resection	14 (15.6%)	9 (23.1%)	3 (7.9%)	2 (15.4%)	0.167
Segmental small bowel resection	7 (8.0%)	2 (5.1%)	5 (13.2%)	0 (0.0%)	0.300
Stoma	3 (3.3%)	2 (5.1%)	1 (2.6%)	0 (0.0%)	1.000
Pouch	4 (4.4%)	1 (2.6%)	2 (5.3%)	1 (7.7%)	0.653
Biochemical parameters					
Haemoglobin (mmol/L)	7.4 ± 0.8	7.2 ± 0.8	7.4 ± 0.9	7.6 ± 1.0	0.332
CRP (mg/L)	3.7 [1.2–9.6]	4.1 [1.0–7.0]	2.7 [1.4–9.1]	3.8 [2.2–12.0]	0.591
FCP (μg/g)	521 [193–1272]	358 [171–1136]	405 [195–1009]	1451 [495–1875]	0.084
TSAT (%)	11.0 [7.0–16.6]	10.5 [6.1–13.3]	10.4 [7.5–15.5]	14.3 [9.5–20.9]	0.305
Ferritin (μg/L)	22 [12–41]	19 [9–36]	21 [14–35]	40 [28–43]	0.038
Hepcidin (μg/mL)	4.12 [0.57–17.11]	4.00 [0.29–9.01]	2.68 [0.44–18.48]	16.82 [11.76–21.91]	0.100
Iron deficiency anaemia	53 (58.9%)	25 (64.1%)	22 (57.9%)	6 (46.2%)	0.455

*Note:* Data are presented as mean ± standard deviation, median [Q1–Q2] or as absolute numbers (%). Continuous variables were compared using a one‐way ANOVA or a Kruskal–Wallis test as appropriate. Categorical data were compared using the *Χ* ^2^‐test. The Benjamini–Hochberg procedure was used to adjust for multiple testing, considering significance under a false discovery rate of 5%. Significant results after correction are indicated in bold; none remained significant after correction.

Abbreviations: CRP, C‐reactive protein; FCP, faecal calprotectin; MTX, Methotrexate; TSAT, transferrin saturation.

^a^ Without biologicals or small molecule therapy.

### 3.2 Hepcidin as a Response Predictor to Iron Therapy

Baseline predictors of response to iron therapy were evaluated, with hepcidin predefined as the primary biomarker of interest. A complete set of iron indices was available for 74 participants at week 5 and 64 participants at week 12, allowing determination of treatment response (Table 2).

**TABLE 2:** Response to iron therapy, stratified by treatment modality.

	Total (*n* = 75)	IV iron (*n* = 36)	Ferrous fumarate (*n* = 30)	Ferric maltol (*n* = 9)	*p*
Response to iron therapy^a^					
Week 5	49/74 (66.2%)	33/36 (91.7%)^^$^	10/29 (34.5%)^^#^	6/9 (66.7%)^$#^	< 0.001
Week 12	39/64 (60.9%)	27/32 (84.4%)^^$^	9/24 (37.5%)^^^	3/8 (37.5%)^$^	< 0.001
Normalisation iron stores^b^					
Week 5	26/74 (35.1%)	25/36 (69.4%)^^$^	0/29 (0.0%)^^#^	1/9 (11.1%)^$#^	< 0.001
Week 12	14/65 (21.5%)	12/33 (36.4%)^^^	2/24 (8.3%)^^^	0/8 (0.0%)	0.011

*Note:* Data are presented as *n*/*N* (%), where *N* represents the number of patients with complete biochemical indices available at the respective time point within each treatment group. Denominators therefore differ between treatment groups and time points due to missing biochemical data. Post hoc testing showed significant pairwise differences between groups marked with the same symbol (#, $, ^).

^a^ Response to iron therapy is defined as an increase > 1.2 mmol/L in haemoglobin or haemoglobin normalisation at week 14 for patients with iron deficiency anaemia; or an increase in ferritin > 100 μg/L and transferrin saturation > 20% at week 14 for patients with iron deficiency without anaemia.

^b^ Ferritin > 100 and TSAT > 20%.

**FIGURE 1:**
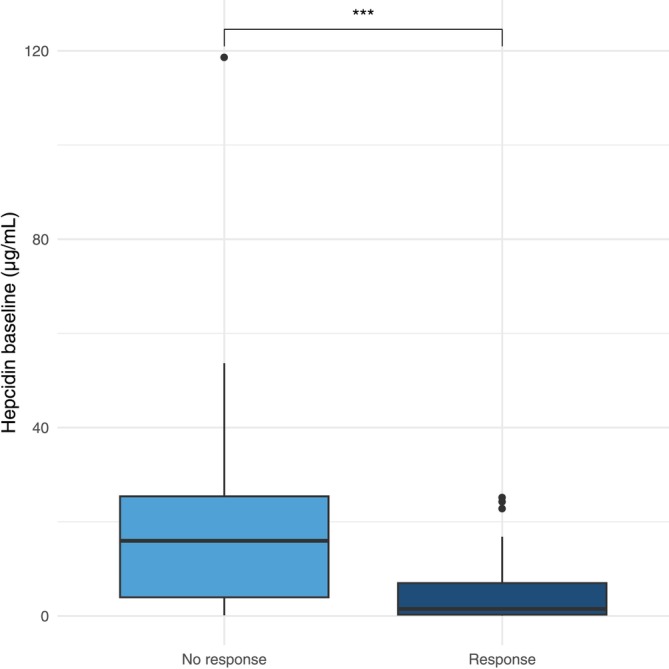
Baseline hepcidin levels in responders and non‐responders. ***p ≤ 0.001.

At baseline, non‐responders had significantly higher ferritin and TSAT values than responders (Table 3), as well as higher hepcidin levels (Figure 1). This difference was most pronounced in the FF group (*p* < 0.01), with similar but non‐significant trends observed in the FM and IV groups (Figure S1). Markers of systemic inflammation, including CRP and IL‐6, did not differ at baseline and remained relatively stable in both responders and non‐responders.

**TABLE 3:** Biochemical parameters at baseline and week 12 for responders and non‐responders.

	Responders (*n* = 39)		*p*	Non‐responders (*n* = 25)		*p*	Δ (baseline‐week 12) responders versus non‐responders	Baseline responders versus non‐responders
	Baseline	Week 12		Baseline	Week 12		*p*	*p*
Systemic iron status parameters								
Haemoglobin (mmol/L)	7.2 ± 0.8	8.5 ± 0.6	**< 0.001**	7.5 ± 0.8	7.6 ± 0.9	0.324	**< 0.001**	0.138
Haemoglobin females	7.1 ± 0.9	8.4 ± 0.6	**< 0.001**	7.3 ± 0.8	7.5 ± 0.8	0.076	**< 0.001**	0.420
Haemoglobin males	7.4 ± 0.6	8.6 ± 0.6	**< 0.001**	7.9 ± 0.7	7.8 ± 1.2	0.669	**< 0.001**	0.047
Ferritin (μg/L)	15.5 [9.5–30.0]	78.0 [33.0–207.0]	**< 0.001**	41.0 [26.5–55.5]	60.0 [47.0–80.0]	**< 0.001**	**0.005**	**< 0.001**
Iron (μmol/L)	7.0 [4.5–9.0]	14.0 [9.3–18.8]	**< 0.001**	10.0 [6.0–13.0]	13.7 [6.0–19.0]	0.077	0.046	0.045
Transferrin (g/L)	3.03 ± 0.51	2.63 ± 0.50	**< 0.001**	2.74 ± 0.50	2.55 ± 0.43	**0.005**	0.032	0.033
TIBC (μmol/L)	75.0 [65.3–83.1]	65.0 [56.3–72.4]	**< 0.001**	69.8 [60.0–74.0]	61.3 [57.0–70.3]	**0.014**	0.090	0.023
TSAT (%)	9.0 [5.7–12.3]	21.6 [13.9–29.2]	**< 0.001**	13.4 [9.7–20.4]	23.9 [10.5–29.2]	0.039	0.043	**0.008**
Hepcidin (μg/mL)	1.49 [0.28–6.98]	23.61 [7.41–39.45]	**< 0.001**	15.96 [3.96–25.40]	15.17 [9.92–30.87]	0.046	**0.001**	**< 0.001**
sTfR (μg/mL)	7.07 [4.90–12.93]	10.42 [8.26–16.21]	**< 0.001**	5.83 [4.31–7.16]	9.50 [8.33–14.20]	**< 0.001**	0.080	0.056
ZPP (ng/mL)	19.78 [14.79–35.49]	25.71 [15.53–45.62]	0.601	17.25 [14.62–22.75]	20.03 [17.68–30.73]	0.190	0.589	0.380
Transferrin/log_10_ (ferritin)	5.6 [4.0–12.1]	6.1 [4.1–9.4]	**< 0.001**	3.9 [2.9–4.6]	5.4 [1.3–7.7]	**< 0.001**	**< 0.001**	**< 0.001**
sTfR/log (ferritin)	2.4 [1.7–5.3]	2.6 [1.8–4.1]	0.134	1.7 [1.3–2.0]	2.3 [1.9–3.3]	**< 0.001**	**< 0.001**	**0.002**
Inflammation‐associated parameters								
CRP (mg/L)	3.3 [0.9–6.0]	2.5 [0.8–6.0]	0.583	4.8 [2.0–12.4]	4.0 [1.5–15.5]	0.842	1.000	0.071
WBC (×10^9^/L)	8.13 [6.32–9.79]	7.20 [5.65–9.79]	0.512	7.69 [6.00–10.7]	7.10 [5.67–8.90]	0.063	0.453	0.880
Platelets (×10^9^/L)	322.2 ± 97.1	282.1 ± 85.8	**< 0.001**	313.2 ± 77.2	294.9 ± 72.4	0.021	0.052	0.700
IL‐6 (pg/mL)	9.48 [2.00–263.34]	5.78 [1.70–409.07]	0.221	3.03 [1.23–5.45]	2.62 [1.23–12.01]	0.070	0.912	0.032
R‐SH (μM)	311.44 [267.75–352.82]	333.96 [297.37–381.16]	**0.005**	325.19 [296.21–354.33]	348.47 [319.58–372.01]	0.045	1.000	0.356
FCP (μg/g)	557 [203–1465]	294 [102–842]	**0.014**	599 [287–985]	394 [87–1231]	0.541	0.235	0.773

*Note:* Data are presented as mean ± standard deviation, median [Q1–Q2] or as absolute numbers (%). For within patient comparisons, paired *t*‐test or Wilcoxon signed‐rank test were used. For comparisons between responders and non‐responders at baseline, unpaired tests were applied (independent *t*‐test or Mann–Whitney U test). Differences in change (Δ) from baseline to 12 weeks between responders and non‐responders were assessed using unpaired tests as appropriate. The Benjamini–Hochberg procedure was used to adjust for multiple testing, considering significance under a false discovery rate of 5%. Values that remained significant after correction are shown in bold.

Abbreviations: CRP, C‐reactive protein; FCP, faecal calprotectin; IL‐6, interleukin‐6; R‐SH, reduced sulfhydryl groups; sTfR, soluble transferrin receptor; TSAT, transferrin saturation; WBC, white blood cell count; ZPP, zinc protoporphyrin.

These baseline differences were reflected in significant associations between several iron‐related biomarkers and treatment response. Each twofold increase in ferritin or hepcidin was associated with a lower chance of response to iron therapy (log_2_(ferritin) OR: 0.38, 95% CI: 0.21–0.69, *p* = 0.002; log_2_(hepcidin) OR: 0.71, 95% CI: 0.56–0.89, *p* = 0.003). Conversely, higher transferrin/log_10_(ferritin) (OR: 2.95, 95% CI: 1.38–6.30, *p* = 0.005) and sTfR/log_10_(ferritin) ratios (OR: 1.29, 95% CI: 1.06–1.59, *p* = 0.015) were associated with increased odds of response (Table S3).

**FIGURE 2:**
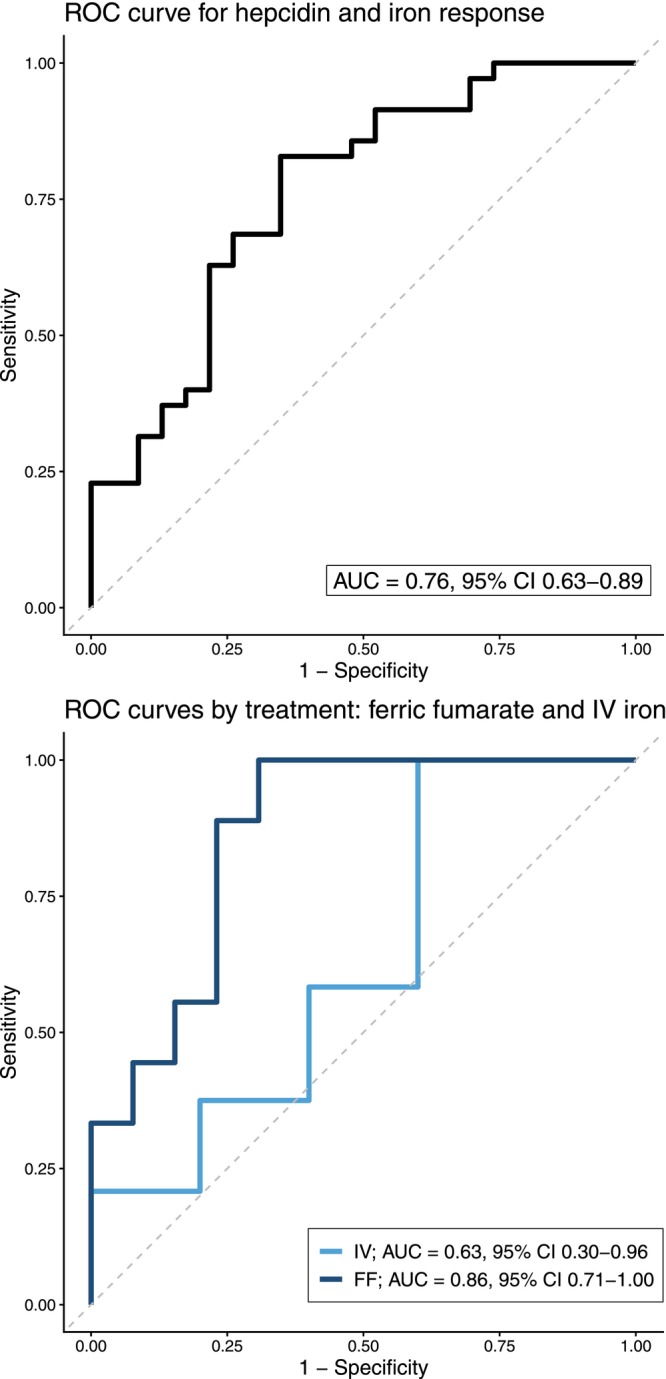
Receiver operating characteristic curves for baseline hepcidin predicting response to iron therapy. Overall analysis and stratified by treatment. AUC, area under the curve; FF, ferrous fumarate; IV, intravenous iron; ROC, receiver operating characteristic curve.

Consistent with these associations, baseline hepcidin demonstrated good discriminative ability for response to iron therapy, with an AUC of 0.76 (95% CI: 0.63–0.89). A hepcidin concentration > 9.20 μg/mL was associated with non‐response, with 83% sensitivity and 65% specificity (Figure 2). When stratified by treatment type, hepcidin showed limited discriminative ability in the IV iron group (AUC: 0.63, 95% CI: 0.30–0.96) but performed well in the FF group (AUC: 0.86, 95% CI: 0.71–1.00). In the FF group, a baseline hepcidin > 2.68 μg/mL indicated a high probability of non‐response, with 89% sensitivity and 77% specificity. Assessment of discriminative ability in the FM group was not possible due to the small number of patients. A complete overview of AUC values is provided in Table S4.

### 3.3 Alternative Biomarkers of Iron Treatment Response

**FIGURE 3:**
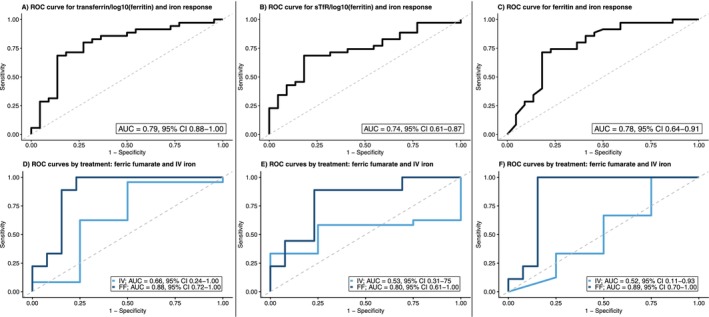
Receiver operating characteristic curves for transferrin/log_10_(ferritin) (A, D), sTfR/log_10_(ferritin) ratio (B, E), and ferritin (C, F) for prediction of response to iron treatment. A–C show overall analyses; D–F show analyses stratified by treatment. AUC, area under the curve; FF, ferrous fumarate; IV, intravenous iron; ROC, receiver operating characteristic curve.

Iron‐related biomarkers demonstrating significant associations with treatment response were examined for their correlations with hepcidin (Table S5). Ferritin, transferrin/log_10_(ferritin) and sTfR/log_10_(ferritin) all showed good discriminative ability in ROC analyses, with AUCs of 0.776, 0.788 and 0.738, respectively. The transferrin/log_10_(ferritin) ratio performed best, with a cut‐off > 2.17 predicting non‐response with 69% sensitivity and 86% specificity (Figure 3). Similar performance was observed for the sTfR/log_10_(ferritin) ratio, while ferritin alone predicted response at values < 23.5 μg/L.

Stratified analyses demonstrated excellent performance in the FF group (AUCs: 0.88, 0.80 and 0.89 for transferrin/log_10_(ferritin), sTfR/log_10_(ferritin) and ferritin, respectively), whereas discriminative ability was limited in the IV group. Overall, transferrin/log_10_(ferritin) and sTfR/log_10_(ferritin) represent promising alternatives to hepcidin for predicting response to oral iron therapy, especially FF. Markers of systemic inflammation (CRP, IL‐6) and intestinal inflammation (FCP) were not associated with treatment response (Table S3).

### 3.4 Response to Iron Therapy

At 12 weeks, 60.9% of patients responded to iron therapy, 39.1% did not respond, and response could not be determined in 11 patients due to missing data (Table 2). Response rates varied significantly by treatment, with the highest response rate observed in the IV group (91.7% at week 5; 84.4% at week 12). In the oral groups, response rates were lower: FF 34.5% and 37.5%, FM 66.7% and 37.5% at weeks 5 and 12, respectively. Normalisation of iron stores at week 12 was achieved in 21.5% overall, with 36.4% in IV, 8.3% in FF and 0% in FM. Among patients with IDA at baseline, haemoglobin increase (≥ 1.2 mmol/L (19.3 g/L)) or normalisation occurred in 73.8% overall, with IV again showing the highest response (90.9%) compared with 53.3% for FF and 60.0% for FM (Table 4). Results for the FM group (*n* = 9) should be interpreted with caution.

**TABLE 4:** Response to iron therapy, stratified by treatment modality, in patients with iron deficiency anaemia at baseline.

	Total (*n* = 49)	IV iron (*n* = 25)	Ferrous fumarate (*n* = 19)	Ferric maltol (*n* = 5)	*p*
≥ 0.6 mmol/L increase haemoglobin					
Week 5	33/48 (68.8%)	20/25 (80.0%)	11/18 (61.1%)	2/5 (40.0%)	0.143
Week 12	26/42 (61.9%)	18/22 (81.8%)^^$^	7/15 (46.7%)^^^	1/5 (20.0%)^$^	0.012
≥ 1.2 mmol/L increase haemoglobin or normalisation					
Week 5	38/48 (79.2%)	23/25 (92.0%)^^^	10/18 (55.6%)^^^	5/5 (100%)	0.007
Week 12	31/42 (73.8%)	20/22 (90.9%)^^$^	8/15 (53.3%)^^^	3/5 (60.0%)^$^	0.029

*Note:* Data are presented as *n*/*N* (%), where *N* represents the number of patients with complete biochemical indices available at the respective time point within each treatment group. Denominators therefore differ between treatment groups and time points due to missing biochemical data. Post hoc testing showed significant pairwise differences between groups marked with the same symbol (#, $, ^).

Biochemical responses differed markedly between responders and non‐responders to iron therapy. Following treatment, responders demonstrated a significant increase in haemoglobin (Δ 1.3 ± 0.8 mmol/L (20.9 ± 12.9 g/L), *p* < 0.001), whereas non‐responders showed no meaningful change (Δ 0.1 ± 0.5 mmol/L (1.6 ± 8.1 g/L), *p* = 0.32). Hepcidin concentrations increased more than 15‐fold in responders (Δ 17.74 μg/mL [7.30–32.43], *p* < 0.001), while remaining largely unchanged in non‐responders. In addition, the sTfR/log_10_(ferritin) and transferrin/log_10_(ferritin) ratios differed significantly between groups: the sTfR/log_10_(ferritin) ratio remained stable in responders but increased over time in non‐responders.

Treatment adherence differed between responders and non‐responders receiving oral iron therapy. High adherence was observed in only 33% of non‐responders, compared with 50% of responders (Figure S2), indicating that non‐responders were more likely to report low adherence to treatment.

### 3.5 Adverse Events

A total of 42 AEs were reported, including two SAEs which were both unrelated to the study intervention (Table S6). The majority of AEs in the oral iron groups were mild gastrointestinal complaints. In the IV iron group, hypophosphatemia was the predominant AE (12.8%). Overall, the safety profile was acceptable and consistent with known risks of the underlying disease and treatment modalities.

## 4 Discussion

This study evaluated whether hepcidin can predict response to oral and IV iron therapy in patients with active IBD receiving immunosuppressive medication. Baseline hepcidin levels emerged as an important determinant of treatment response, with higher concentrations associated with poorer outcomes, especially following oral iron therapy. In parallel, ferritin‐based indices, particularly the transferrin/log_10_(ferritin) ratio, also demonstrated strong predictive performance, providing a practical alternative when direct hepcidin measurement is not available.

Patients with hepcidin levels > 9.20 μg/mL had a low probability of response irrespective of the route of administration. Interestingly, a lower cut‐off of 2.68 μg/mL identified non‐responders to FF with high sensitivity and specificity. This implicates that patients with mild to moderate disease activity and mild ID(A) are more likely to benefit from IV iron than from oral iron therapy. Mechanistically, elevated hepcidin levels limit intestinal iron absorption by downregulating ferroportin, thereby reducing the efficacy of oral iron supplementation. Moreover, previous work has demonstrated that oral iron supplementation itself can further stimulate hepcidin production, resulting in reduced fractional iron absorption, particularly in inflammatory states. IV iron formulations circumvent intestinal iron absorption but do not fully bypass the hepcidin‐ferroportin axis. Following IV administration, iron is taken up by macrophages and its release remains regulated by hepcidin. Consequently, elevated hepcidin levels, driven by ongoing inflammation, may therefore still lead to a suboptimal response to IV iron therapy [16, 34]. In line with these findings, the transferrin/log_10_(ferritin) ratio also demonstrated strong predictive performance, offering a practical alternative marker for identifying likely responders.

Although hepcidin is classically upregulated by inflammation, emerging evidence suggests that absolute iron availability may be the dominant determinant of circulating hepcidin levels, resulting in a more nuanced relationship between systemic inflammation and iron homeostasis [35]. Previous studies have shown that hepcidin can discriminate between absolute ID and anaemia of inflammation in patients with active IBD. In this context, hepcidin correlates more strongly with iron parameters than with inflammatory markers, and the effect of inflammation on hepcidin is modest once absolute ID is present [5, 14, 16, 17, 36]. This was also observed in the current study, where classical inflammatory markers were not predictive of treatment response. In addition, immunosuppressive therapies commonly used in IBD may further suppress hepcidin production, potentially enhancing iron absorption and mobilisation [16, 37]. Consistent with these findings, a paediatric IBD cohort demonstrated that prognostic models incorporating baseline hepcidin outperformed traditional iron indices in identifying children unlikely to respond to iron supplementation [13].

When examining treatment outcomes in this cohort, only 21.5% of patients achieved full normalisation of iron stores by week 12, despite 73.8% showing a meaningful improvement in haemoglobin. This suggests that haemoglobin recovery precedes the replenishment of iron stores, reflecting the physiological prioritisation of erythropoiesis over restoration of tissue iron [38]. This distinction is clinically important, as treatment strategies focused solely on haemoglobin normalisation may fail to adequately restore iron stores, thereby predisposing patients to recurrent anaemia. A survey among gastroenterologists has shown that, in routine clinical practice, treatment decisions are often guided primarily by haemoglobin levels rather than by markers of iron stores, resulting in systematic undertreatment of ID in IBD [39]. Moreover, a decline in response rates between weeks 5 and 12 was observed across all treatment groups. This could reflect unrecognised iron losses or demand leading to either underestimation of the required replacement dose or incomplete administration of the recommended IV iron due to logistic challenges.

Several limitations should be considered when interpreting these findings. First, this analysis is based on pooled data from two clinical trials. Although the study protocols were largely comparable, important differences existed. Most notably, dosing of FF was not uniform across studies: while patients from both studies started with the same doses, FF dosing was adjusted according to haemoglobin levels after 4 or 12 weeks in the OVI‐IBD study, whereas dosing remained unchanged throughout the PRIme trial. In addition, FM was prescribed exclusively in the PRIme study, resulting in a small sample size that was underpowered for predictive analyses. This, together with the relatively small number of patients in individual subgroups, limits the robustness of subgroup‐specific analyses. Second, adherence to oral iron therapy was limited, whereby suboptimal treatment response may partly reflect poor adherence rather than response to oral iron.

The novel biomarkers hepcidin and sTfR are promising tools for guiding iron therapy. However, they are not yet routinely implemented due to methodological limitations, including the lack of assay standardisation and validated reference ranges for hepcidin, as well as platform‐dependent variability in sTfR measurements [15, 34, 40]. Importantly, the present study indicates that accurate prediction of treatment response does not necessarily require direct measurement of hepcidin or sTfR. More widely available ferritin‐based indices, particularly the transferrin/log_10_(ferritin) ratio, perform comparably. Given the invasive nature and limited feasibility of bone marrow iron staining, which precludes its routine use despite being the historical gold standard, these findings underscore the need for validated, non‐invasive alternatives. While hepcidin offers mechanistic insight, readily available and cost‐effective markers are sufficient to guide personalised iron therapy in most clinical settings. Future studies should focus on assay standardisation and further validation of transferrin/log_10_(ferritin) as a practical tool for routine decision‐making.

Predicting response to iron therapy can ensure timely and adequate correction of ID(A), improve patient‐reported outcomes, and reduce unnecessary or ineffective treatment, thereby optimising healthcare resource use. Baseline hepcidin can identify patients less likely to respond to iron, particularly oral therapy, while ferritin‐based indices, such as the transferrin/log_10_(ferritin) ratio, offer practical alternatives. These findings support a personalised approach to iron supplementation in IBD.

## Author Contributions

**Roberta Loveikyte:** conceptualization, methodology, investigation, resources, data curation, project administration, writing – review and editing. **Sander van der Marel:** resources. **Lola J. M. Koppelman:** conceptualization, methodology, formal analysis, investigation, resources, data curation, writing – original draft, visualization, project administration. **Andrea E. van der Meulen‐de Jong:** conceptualization, methodology, investigation, resources, writing – review and editing, supervision, funding acquisition. **Rogier L. Goetgebuer:** resources. **Annemarie C. de Vries:** resources. **Gerard Dijkstra:** conceptualization, methodology, resources, supervision, funding acquisition.

## Funding

This study is comprised of two investigator‐initiated studies. The OVI‐IBD study was supported by ZonMw, the Netherlands Organisation for Health Research and Development (grant number 10140022010008). The PRIme study has been partially funded by Norgine Ltd. In addition, Norgine Ltd. has provided ferric maltol for this study at no cost. ZonMw and Norgine Ltd. had no role in the design of the study, data collection, analysis, interpretation or writing of the manuscript.

## Conflicts of Interest

R.L. has received travel expenses and speaker's fees from Galapagos and Cablon Medical and served on the advisory board at Cablon Medical. R.L.G. has received speakers fees by Janssen Cilag BV, Takeda, Pfizer and Celltrion. A.C.V. serves in the advisory boards for Takeda, Janssen, AbbVie, Pfizer, Galapagos/Alfa Sigma and has received unrestricted research grants from Takeda, Lilly, Janssen, Pfizer, MSD, AbbVie; G.D. has received speakers fees from AbbVie and Astra‐Zeneca. A.E.M.J. has received advisory board honoraria and speaker fees from Janssen, Takeda, Galapagos, Vedanta, Ferring, AbbVie and Pfizer, research grants from Norgine, Cablon, AlfaSigma and ZonMw, and an unrestricted grant from Roba Metals B.V.

## Supporting information


**Figure S1:** Baseline hepcidin levels responders vs. non‐responders per treatment group. **p ≤ 0.01.
**Figure S2:**. Adherence levels by treatment response in percentages.
**Figure S3:**. Overview of the study inclusions.
**Table S1:**. Extensive overview of baseline characteristics.
**Table S2:**. Missing data.
**Table S3:**. Univariable logistic regression analyses of baseline predictors of response to iron therapy.
**Table S4:**. Predictive performance (AUC, sensitivity, specificity) of iron biomarkers.
**Table S5:**. Spearman correlation coefficients between hepcidin and iron‐related biomarkers.
**Table S6:**. Overview of (serious) adverse events.
**Table S7:**. Response stratified by iron formulation.
**Table S8:**. Response to iron therapy, stratified by treatment modality, inpatients with iron deficiency anaemia at baseline.
**Table S9:**. Sensitivity and detection performance of ELISA assays.

## Data Availability

The data that support the findings of this study are available on request from the corresponding author. The data are not publicly available due to privacy or ethical restrictions.
