# Supplementary material for: Clinical Trial: Predicting Response to Iron Therapy in Patients With Active Inflammatory Bowel Disease Using Hepcidin and Functional Iron Indices: A Multicentre Randomised Trial
Source: Aliment Pharmacol Ther. 2026 Jun 8;64(2):166–76. doi: 10.1111/apt.70775 (PMC13309206; doi:10.1111/apt.70775)
Supplement: Supplementary file 1 — Figure S1: Baseline hepcidin levels responders vs. non‐responders per treatment group. **p ≤ 0.01. Figure S2:. Adherence levels by treatment response in percentages. Figure S3:. Overview of the study inclusions. Table S1:. Extensive overview of baseline characteristics. Table S2:. Missing data. Table S3:. Univariable logistic regression analyses of baseline predictors of response to iron therapy. Table S4:. Predictive performance (AUC, sensitivity, specificity) of iron biomarkers. Table S5:. Spearman correlation coefficients between hepcidin and iron‐related biomarkers. Table S6:. Overview of (serious) adverse events. Table S7:. Response stratified by iron formulation. Table S8:. Response to iron therapy, stratified by treatment modality, inpatients with iron deficiency anaemia at baseline. Table S9:. Sensitivity and detection performance of ELISA assays. [file APT-64-166-s001.docx]

Appendix 1

**Dutch iron study group:**

Dr. M. Duijvestein^1^, dr. M. van der Have^2^, dr. C. Horjus^3^, Dr. W.G.N. Mares^4^, Dr. Z. Mujagic^5^, Prof. Dr. B Oldenburg^6^, dr. R. West^7^

*^1^Department of Gastroenterology and Hepatology, Radboud University Medical Center, Nijmegen, The Netherlands*

*^2^Department of Gastroenterology and Hepatology, Admiraal De Ruyter Hospital, Goes, The Netherlands*

*^3^Department of Gastroenterology and Hepatology, Rijnstate Hospital, Arnhem, The Netherlands*

*^4^Department of Gastroenterology and Hepatology, Ziekenhuis Gelderse Vallei, Ede, The Netherlands*

*^5^Department of Gastroenterology and Hepatology, Maastricht University Medical Center+, Maastricht, The Netherlands*

*^6^Department of Gastroenterology and Hepatology, University Medical Center Utrecht, Utrecht, The Netherlands*

*^7^Department of Gastroenterology and Hepatology, Franciscus Gasthuis & Vlietland, Rotterdam, The Netherlands*

Appendix 2

Supplementary Table 1. Extensive overview of baseline characteristics

|  | **Total  (n=90)** | **IV Iron  (n=39)** | **Ferrous fumarate (n=38)** | **Ferric maltol  (n=13)** | **p-value** |
| --- | --- | --- | --- | --- | --- |
| Age (years) | 40.0 [30.0–56.8] | 40.0 [26.5–57.5] | 44.5 [30.25–59.25] | 38.0 [24.0–42.0] | 0.411 |
| Female sex | 53 (58.9%) | 22 (56.4%) | 22 (57.9%) | 9 (69.2%) | 0.707 |
| Disease duration (years) | 10.0 [3.0–17.8] | 9.0 [3.5–17.5] | 10.5 [3.0–18.25] | 12.0 [8.0–15.0] | 0.785 |
| Age at diagnosis |  |  |  |  |  |
| < 17 years | 15 (16.7%) | 6 (15.4%) | 4 (10.5%) | 5 (38.5%) | 0.063 |
| 17-40 years | 57 (63.3%) | 24 (61.5%) | 26 (68.4%) | 7 (53.8%) | 0.612 |
| >40 years | 18 (20.0%) | 9 (23.1%) | 8 (21.1%) | 1 (7.7%) | 0.575 |
| Crohn’s Disease | 55 (57.8%) | 26 (66.7%) | 22 (57.9%) | 4 (30.8%) | 0.076 |
| Disease location |  |  |  |  |  |
| Terminal ileum | 18 (34.6%) | 12 (46.2%) | 5 (22.7%) | 1 (25.0%) | 0.216 |
| Colon | 12 (23.1%) | 3 (11.5%) | 8 (36.4%) | 1 (25.0%) | 0.126 |
| Ileocolonic | 22 (42.3%) | 11 (42.3%) | 9 (40.9%) | 2 (50.0%) | 0.944 |
| Upper GI-involvement ^†^ | 7 (13.5%) | 3 (11.5%) | 3 (13.6%) | 1 (25.0%) | 0.763 |
| Perianal disease ^†^ | 15 (28.8%) | 6 (23.1%) | 9 (40.9%) | 0 (0.0%) | 0.165 |
| Behaviour |  |  |  |  |  |
| inflammatory | 32 (61.5%) | 16 (61.5%) | 16 (72.7%) | 0 (0.0%) | 0.023 |
| Stricturing | 19 (36.5%) | 9 (34.6%) | 6 (27.3%) | 4 (100.0%) | 0.020 |
| Penetrating | 6 (11.5%) | 5 (19.2%) | 1 (4.5%) | 0 (0.0%) | 0.214 |
| Ulcerative Colitis | 38 (42.2%) | 13 (33.3%) | 16 (42.1%) | 9 (69.2%) | 0.076 |
| Disease extension |  |  |  |  |  |
| Proctitis | 4 (10.5%) | 1 (7.7%) | 3 (18.8%) | 0 (0.0%) | 0.378 |
| Left-sided colitis | 7 (18.4%) | 2 (15.4%) | 2 (12.5%) | 3 (33.3%) | 0.410 |
| Pancolitis | 27 (71.1%) | 10 (76.9%) | 11 (68.8%) | 6 (66.7%) | 0.842 |
| Medication use |  |  |  |  |  |
| No medication | 3 (3.3%) | 2 (5.1%) | 0 (0.0%) | 1 (8.33%) | 0.291 |
| Corticosteroids ^‡^ | 8 (8.9%) | 4 (10.3%) | 3 (7.9%) | 1 (7.7%) | 0.933 |
| Aminosalicylates ^‡^ | 7 (7.8%) | 2 (5.3%) | 2 (5.3%) | 3 (23.1%) | 0.012 |
| thiopurines / MTX ^‡^ | 2 (2.2%) | 1 (2.6%) | 1 (2.6%) | 0 (0.0%) | 0.839 |
| biologicals/Small molecules | 75 (8.3%) | 33 (84.6%) | 33 (8.7%) | 8 (61.5%) | 0.069 |
| History of surgery | 26 (28.9%) | 13 (33.3%) | 10 (26.3%) | 3 (23.1%) | 0.701 |
| (partial) colectomy | 7 (7.8%) | 2 (5.1%) | 4 (10.5%) | 1 (7.7%) | 0.765 |
| Ileocecal resection | 14 (15.6%) | 9 (23.1%) | 3 (7.9%) | 2 (15.4%) | 0.167 |
| Segmental small bowel resection | 7 (8.0%) | 2 (5.1%) | 5 (13.2%) | 0 (0.0%) | 0.300 |
| Stoma | 3 (3.3%) | 2 (5.1%) | 1 (2.6%) | 0 (0.0%) | 1.000 |
| pouch | 4 (4.4%) | 1 (2.6%) | 2 (5.3%) | 1 (7.7%) | 0.653 |
| Biochemical parameters |  |  |  |  |  |
| MCV (fL) | 85.4 [81.0–89.8] | 84.1 [ 78.0–88.0] | 85.3 [81.9–90.0] | 86.0 [84.8–91.3] | 0.410 |
| Albumin (g/L) | 40.7 ± 4.6 | 39.7 ± 4.3 | 41.4 ± 4.3 | 41.5 ± 6.1 | 0.245 |
| Haematocrit (L/L) | 0.372 ± 0.033 | 0.366 ±0.029 | 0.375 ± 0.035 | 0.383 ± 0.038 | 0.211 |
| Creatinine (µmol/l) | 70.0 [62.0–79.0] | 71.0[61.0–86.0] | 69.0[64.0–77.0] | 67.0[58.0–76.0] | 0.584 |
| eGFR (ml/min/1,73 m^2^) | 90 [83.0–90.0] | 90.0 [78.0–90.0] | 90 [84.0–90.0] | 90 [90.0–98.0] | 0.151 |
| Folic acid (nmol/L) | 14.7 [10.5–23.1] | 14.0[9.9–22.1] | 17.7[10.9–23.0] | 13.2 [9.1–32.9] | 0.579 |
| Vitamin B12 (pmol/L) | 307 [235–433] | 324 [246–433] | 284 [225–400] | 383 [253–486] | 0.570 |
| Vitamin D (nmol/L) | 57.5 [41.0–69.0] | 61.0 [42.0–71.0] | 56.9 [43.5–66.4] | 42.5 [34.5–55.5] | 0.433 |
| Haemoglobin (mmol/L) | 7.4 ± 0.8 | 7.2 ± 0.8 | 7.4 ± 0.9 | 7.6 ± 1.0 | 0.332 |
| CRP (mg/L) | 3.7 [1.2–9.6] | 4.1 [1.0–7.0] | 2.7 [1.4–9.1] | 3.8 [2.2–12.0] | 0.591 |
| FCP (µg/g) | 521 [193–1272] | 358 [171–1136] | 405 [195–1009] | 1451 [495–1875] | 0.084 |
| TSAT (%) | 11.0[7.0–16.6] | 10.5[6.1–13.3] | 10.4[7.5–15.5] | 14.3[9.5–20.9] | 0.305 |
| Ferritin (µg/L) | 22 [ 12–41] | 19 [ 9–36] | 21[14–35] | 40 [ 28–43] | 0.038 |
| Iron (µmol/L) | 7.7 [ 5.0–11.1] | 7.6 [4.9–10.0] | 7.4[5.8–12.4] | 9.0[6.0–13.0] | 0.450 |
| Transferrin (g/L) | 2.86±0.53 | 2.9 ± 0.5 | 2.9 ± 0.6 | 2.6 ± 0.4 | 0.218 |
| Phosphate (mmol/L) | 1.00±0.19 | 1.00 ± 0.17 | 1.03 ± 0.2 | 0.94 ± 0.22 | 0.396 |
| TIBC (µg/dL) | 71.4±13.3 | 71.7 ± 13.6 | 73.2 ± 13.8 | 65.7 ± 9.3 | 0.218 |
| Hepcidin (µg/mL) | 4.12 [0.57–17.11] | 4.00 [0.29–9.01] | 2.68 [0.44–18.48] | 16.82 [11.76–21.91] | 0.100 |
| sTfR (µg/mL) | 6.61 [4.90–9.27] | 7.01 [5.05–10.84] | 6.49 [5.00–9.21] | 6.41 [ 3.92–7.10] | 0.486 |
| IL-6 (pg/mL) | 4.27 [1.88–118.11] | 9.48 [2.20–378.96] | 3.78 [2.81–32.69] | 1.18 [0.94–2.30] | 0.016 |
| R-SH (µM) | 321.80 [288.38–355.43] | 307.07 [270.67–353.37] | 328.62 [301.75–355.69] | 354.24 [327.60–370.96] | 0.157 |
| ZPP (ng/mL) | 17.67 [14.38–31.51] | 20.96 [14.62–35.97] | 17.53 [13.91–22.22] | 16.76 [13.11–24.97] | 0.670 |
| Erythropoietin (pg/mL) | 162.94 [133.07–349.97] | 170.69 [133.14–350.22] | 203.23 [150.18–302.00] | 140.52 | 0.794 |
| *Data are presented as mean ± standard deviation, median [Q1–Q2] or as absolute numbers (%). Continuous variables were compared using a one-way ANOVA or a Kruskal Wallis test as appropriate. Categorical data were compared using the Χ2-test. † Upper GI-involvement (Montreal L4%) and perianal disease are presented as a modifier, which includes upper GI-involvement or perianal disease in addition to other disease locations. ‡ Without biologicals or small molecule therapy. The Benjamini–Hochberg procedure was used to adjust for multiple testing, considering significance under a false discovery rate of 5%. Values that remained significant after correction are shown in bold. Abbreviations: CRP, C-reactive protein; FCP, faecal calprotectin; IL-6, interleukin-6; MTX, Methotrexate; R-SH, reduced sulfhydryl groups; sTfR, soluble transferrin receptor; TSAT, transferrin saturation; WBC, white blood cell count; ZPP, zinc protoporphyrin.* | | | | | |

Supplementary Table 2. Missing data

|  | **Baseline (n=75)** | **Week 5 (n=75)** | **Week 12 (n=75)** |
| --- | --- | --- | --- |
| Haemoglobin | 1 (1.3%) | 1 (1.3%) | 10 (13.3%) |
| Haematocrit | 2 (2.7%) | 1 (1.3%) | 10 (13.3%) |
| MCV | 1 (1.3%) | 1 (1.3%) | 10 (13.3%) |
| Erythropoietin | 59 (78.7%) | — | 39 (52.0%) |
| Iron | 1 (1.3%) | 3 | 11 (14.7%) |
| Ferritin | 2 (2.7%) | 2 (2.7%) | 9 (12.0%) |
| TSAT | 1 (1.3%) | 3 (4.0%) | 8 (10.7%) |
| Transferrin | 1 (1.3%) | 4 (5.3%) | 10 (13.3%) |
| TIBC | 1 (1.3%) | — | 11 (14.7%) |
| Hepcidin | 7 (9.3%) | — | 10 (13.3%) |
| sTfR | 5 (6.7%) | — | 10 (13.3%) |
| ZPP | 5 (6.7%) | — | 10 (13.3%) |
| CRP | 2 (2.7%) | 3 (4.0%) | 9 (12.0%) |
| FCP | 3 (4.0%) | — | 20 (26.7%) |
| IL-6 | 12 (16.0%) | — | 13 (17.3%) |
| Creatinine | 1 (1.3%) | — | — |
| eGFR | 1 (1.3%) | — | — |
| Phosphate | 4 (5.3%) | 4 (5.3%) | 11 (14.7%) |
| Albumin | 6 (8.0%) | — | — |
| Folic acid | 7 (9.3%) | — | — |
| Vitamin B12 | 7 (9.3%) | — | — |
| Vitamin D | 15 (20.0%) | — | — |
| R-SH | 5 (6.7%) | — | 11 (14.7%) |
| *Data are presented as absolute numbers (%). Abbreviations: CI, Confidence Interval; CRP, C-reactive protein; FCP, faecal calprotectin; IL-6, interleukin-6; OR, Odds Ratio R-SH, reduced sulfhydryl groups; sTfR, soluble transferrin receptor; TSAT, transferrin saturation; WBC, white blood cell count; ZPP, zinc protoporphyrin.* | | | |

Supplementary Table 3. Univariable logistic regression analyses of baseline predictors of response to iron therapy

|  | **OR (95% CI)** | **P–value** |
| --- | --- | --- |
| Ferritin (µg/L) | 0.943 (0.911–0.977) | **<0.001** |
| log₂(ferritin) (µg/L) | 0.375 (0.205–0.687) | **0.002** |
| Hepcidin (µg/mL) | 0.907 (0.851–0.966) | **0.002** |
| log₂(hepcidin) (µg/mL) | 0.707 (0.563–0.888) | **0.003** |
| transferrin/log_10_(ferritin) | 2.953 (1.384–6.300) | **0.005** |
| sTfR/log_10_(ferritin) | 1.292 (1.051–1.589) | **0.015** |
| TIBC (µg/dL) | 1.053 (1.005–1.104) | **0.029** |
| Transferrin (g/L) | 3.662 (1.142–11.742) | **0.029** |
| sTfR (µg/mL) | 1.170 (1.003–1.363) | **0.045** |
| TSAT (%) | 0.948 (0.891–1.007) | 0.085 |
| Haemoglobin (mmol/L) | 0.594 (0.300–1.175) | 0.135 |
| Iron (µmol/L) | 0.946 (0.873–1.024) | 0.170 |
| CRP (mg/L) | 0.958 (0.897–1.024) | 0.211 |
| IL-6 (pg/mL) | 1.006 (0.997–1.014) | 0.217 |
| R-SH (µM) | 0.994 (0.985–1.004) | 0.242 |
| FCP (µg/g) | 1.000 (0.992–10.003) | 0.323 |
| Erythropoietin (pg/mL) | 1.001 (0.996–1.007) | 0.680 |
| ZPP (ng/mL) | 1.002 (0.989–1.015) | 0.761 |
| MCV (fL) | 0.987 (0.841–1.158) | 0.872 |
| *Odds ratios and 95% CIs were derived from separate univariate logistic regression models. Response to iron therapy is defined as an increase >1.2 mmol/l in haemoglobin or haemoglobin normalization at week 14 for patients with iron deficiency anaemia; or an increase in ferritin >100 μg/l%) and transferrin saturation >20% at week 14 for patients with iron deficiency without anaemia. Abbreviations: CI, Confidence Interval; CRP, C-reactive protein; FCP, faecal calprotectin; IL-6, interleukin-6; OR, Odds Ratio R-SH, reduced sulfhydryl groups; sTfR, soluble transferrin receptor; TSAT, transferrin saturation; WBC, white blood cell count; ZPP, zinc protoporphyrin.* | | |

Supplementary Table 4. Predictive performance (AUC, sensitivity, specificity) of iron biomarkers

| Variable | AUC | 95% CI | Cut-off | Sensitivity | Specificity |
| --- | --- | --- | --- | --- | --- |
| Hepcidin | 0.76 | 0.63–0.89 | >9.20 µg/mL | 83% | 65% |
| *Intravenous Iron* | 0.63 | 0.30–0.96 | >2.47 µg/mL | 96% | 40% |
| *Ferrous Fumarate* | 0.86 | 0.71–1.00 | >2.68 µg/mL | 89% | 77% |
| Transferrin/log_10_(ferritin) | 0.79 | 0.88–1.00 | >2.17 | 69% | 86% |
| *Intravenous Iron* | 0.66 | 0.24–1.00 | >1.34 | 96% | 50% |
| *Ferrous Fumarate* | 0.88 | 0.72–1.00 | >2.08 | 100% | 77% |
| sTfR/log_10_(ferritin) | 0.74 | 0.61–0.87 | >4.58 | 69% | 82% |
| *Intravenous Iron* | 0.53 | 0.31–0.75 | >4.52 | 58% | 75% |
| *Ferrous Fumarate* | 0.80 | 0.61–1.00 | >4.56 | 89% | 77% |
| Ferritin | 0.78 | 0.64–0.91 | <23.5 µg/L | 71% | 82% |
| *Intravenous Iron* | 0.52 | 0.11–0.93 | <79.0 µg/L | 100% | 25% |
| *Ferrous Fumarate* | 0.89 | 0.70–1.00 | <23.5 µg/L | 100% | 85% |
| *Cut-off values were derived using the Youden index. Sensitivity and specificity are reported at the optimal cut-off point for each biomarker. Abbreviations: AUC, area under the curve; sTfR, soluble transferrin receptor.* | | | | | |

Supplementary Table 5. Spearman correlation coefficients between hepcidin and iron-related biomarkers.

|  | **Spearman’s ρ** | **p-value** |
| --- | --- | --- |
| Ferritin | 0.715 | **<0.001** |
| TSAT | 0.419 | **<0.001** |
| sTfR | -0.344 | **0.004** |
| sTfR/log_10_(ferritin) | -0.592 | **<0.001** |
| Transferrin/log_10_(ferritin) | -0.722 | **<0.001** |
| *Abbreviations: sTfR, soluble transferrin receptor; TSAT, transferrin saturation* | | |

Supplementary Table 6. Overview of (serious) adverse events

|  | **Adverse events** | | |
| --- | --- | --- | --- |
|  | **IV Iron (n=39)** | **Ferrous fumarate (n=38)** | **Ferric maltol (n=13)** |
| **Serious Adverse Events** |  |  |  |
| Hospitalisation |  |  |  |
| *Exacerbation of eGPA* | 1 (2.6%) |  |  |
| *Severe rectal bleeding in Crohn’s disease* | 1 (2.6%) |  |  |
| **Adverse Events** |  |  |  |
| Infusion reaction | 1 (2.6%) |  |  |
| Hypophosphatemia | 5 (12.8%) |  |  |
| IBD exacerbation |  |  |  |
| *Mild exacerbation* *^†^* | 1 (2.6%) |  | 1 (7.69%) |
| *Moderate-to-severe exacerbation* *^‡^* |  | 2 (5.26%) |  |
| *Azathioprine-related liver test   abnormalities* | 1 (2.6%) |  |  |
| *Azathioprine side effects after brand  switch* | 1 (2.6%) |  |  |
| Gastrointestinal complaints not related to IBD activity |  |  |  |
| *Nausea* |  | 4 (10.53%) |  |
| *Diarrhoea* |  | 1 (2.63%) | 1 (7.69%) |
| *Flatulence* |  | 1 (2.63%) | 1 (7.69%) |
| *Abdominal pain* |  | 2 (5.26%) | 2 (15.38%) |
| *Constipation* |  | 2 (5.26%) |  |
| *Tarry stool* |  | 3 (7.89%) | 2 (15.38%) |
| Other complaints |  |  |  |
| *Hyperventilation episode* |  | 2 (5.26%) |  |
| *Seton placement* |  | 1 (2.63%) |  |
| *Ankle fracture* |  | 1 (2.63%) |  |
| *Urticaria* | 1 (2.6%) |  |  |
| *Gastrointestinal infections* | 2 (5.13%) | 1 (2.63%) |  |
| *Arthralgia* | 1 (2.6%) |  |  |
| *Muscle pain* | 1 (2.6%) |  |  |
| **TOTAL COUNT OF EVENTS** | **16** | **19** | **7** |
| *Data are presented as absolute numbers (%).† Includes clinical, biochemical, endoscopic, or radiologic disease activity that was treated locally or by optimizing levels of systemic medication. ‡ Includes clinical, biochemical, endoscopic, or radiologic disease activity that necessitated changing systemic therapy.* | | | |

Supplementary Table 7. Response stratified by iron formulation

|  |  | **Total**  **(n=75)** | **IV iron  (n=36)** | **Ferrous Fumarate (n=30)** | **Ferric Maltol (n=9)** | **p-value** |
| --- | --- | --- | --- | --- | --- | --- |
| **Response to iron therapy** **^†^** | **week 5** | 49 (65.3%) | 33 (91.7%)^^$^ | 10 (33.3%)^^#^ | 6 (66.7%)^$#^ | **<0.001** |
|  | **week 12** | 39 (52.0%) | 27 (75.0%)^^$^ | 9 (30.0%)^^^ | 3 (33.3%)^$^ | **<0.001** |
| **Normalisation Iron Stores ^‡^** | **week 5** | 26 (34.7%) | 25 (69.4%)^^$^ | 0 (0.0%)^^#^ | 1 (11.1%)^$#^ | **<0.001** |
|  | **week 12** | 14 (18.7%) | 12 (33.3%)^^^ | 2 (6.7%)^^^ | 0 (0.0%) | **0.011** |
| *Data are presented as absolute numbers (%).Percentages are calculated using the total (n = 75); response status was unknown in 1 patient at week 5 and 11 patients at week 12 due to missing biochemical data. Post-hoc testing showed significant pairwise differences between groups marked with the same symbol (#,$,^). †Response to iron therapy is defined as an increase >1.2 mmol/l in haemoglobin or haemoglobin normalization at week 14 for patients with iron deficiency anaemia; or an increase in ferritin >100 μg/l) and transferrin saturation >20% at week 14 for patients with iron deficiency without anaemia].* *‡ Ferritin > 100 and TSAT >20%.* | | | | | | |

Supplementary Table 8. Response to iron therapy, stratified by treatment modality, inpatients with iron deficiency anaemia at baseline.

|  |  | **Total (n=49)** | **IV iron  (n=25)** | **Ferrous Fumarate (n=19)** | **Ferric Maltol (n=5)** | **p-value** |
| --- | --- | --- | --- | --- | --- | --- |
| **≥0.6 mmol/l increase haemoglobin** | **week 5** | 33 (67.3%) | 20 (80.0%) | 11 (57.9%) | 2 (40.0%) | 0.143 |
|  | **week 12** | 26 (53.1%) | 18 (81.1%)^^$^ | 7 (46.7%)^^^ | 1 (20.0%)^$^ | 0.012 |
| **≥1.2 mmol/l increase haemoglobin or normalisation** | **week 5** | 38 (77.6%) | 23 (92.0%)^^^ | 10 (52.6%)^^^ | 5 (100%) | 0.007 |
|  | **week 12** | 31 (63.3%) | 20 (80.0%)^^$^ | 8 (42.1%)^^^ | 3 (60.0%)^$^ | 0.029 |
| *Data are presented as absolute numbers (%)*.*Percentages are calculated using the total (n = 49); response status was unknown in 1 patient at week 5 and 7 patients at week 12 due to missing biochemical data.* *Post-hoc testing showed significant pairwise differences between groups marked with the same symbol (#,$,^).* | | | | | | |

Supplementary Table 9. Sensitivity and detection performance of ELISA assays

| **Biomarker** | **Assay range** | **Detection rate (%)** | **Extrapolated values (%)** |
| --- | --- | --- | --- |
| Hepcidin (pg/mL) | 15.60–1000.00 | 100.00% | 3.18% |
| sTfR (µg/mL) | 0.05–2.00 | 100.00% | 0.64% |
| IL-6 (pg/mL) | 0.20–600.00 | 98.09% | 11.46% |
| EPO (pg/mL) | 31.20–2000.00 | 35.67% | 0.00% |
| ZPP (ng/mL) | 0.50–200.00 | 100.00% | 8.28% |
| R-SH (µM) | 15.625–1000.000 | 100.00% | 0.00% |

*LLoD: lower limit of detection; ULoD: upper limit of detection. sTfR: soluble Transferrin Receptor, IL-6: Interleukin 6, EPO: erythropoietin, R-SH: free thiols.*


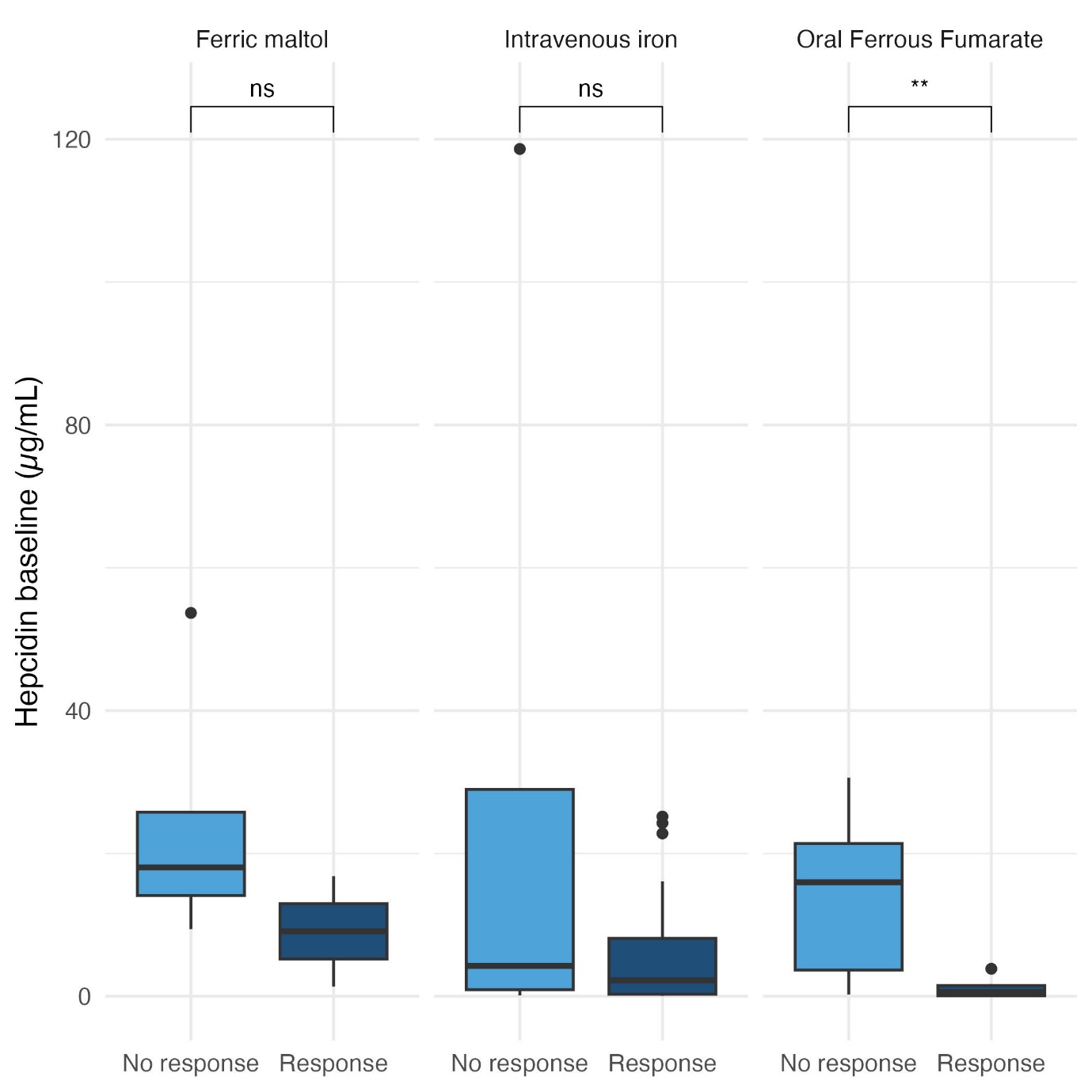


Supplementary Figure 1. Baseline hepcidin levels responders vs. non-responders per treatment group. **P ≤ 0.01


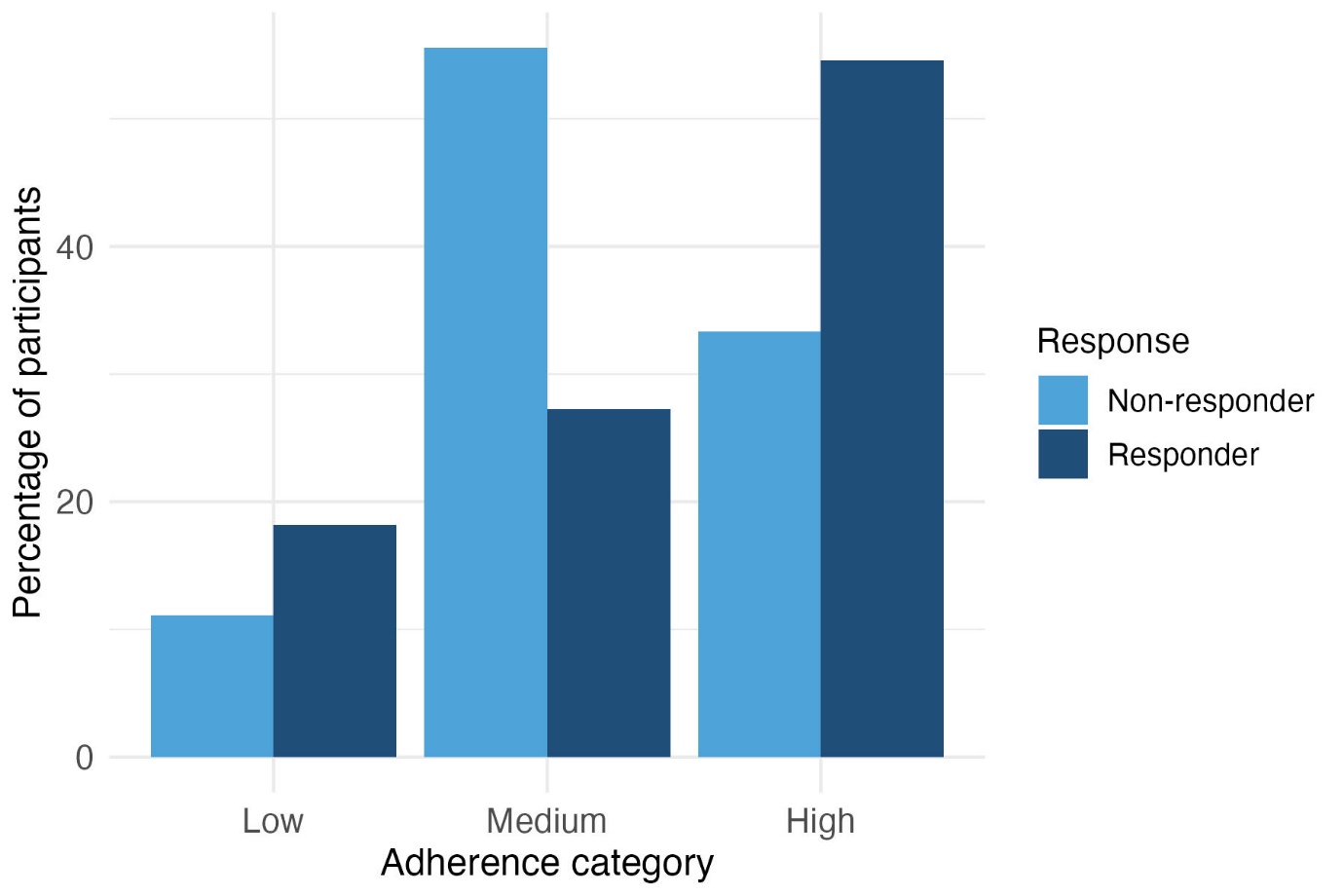


Supplementary Figure 2. Adherence levels by treatment response in percentages


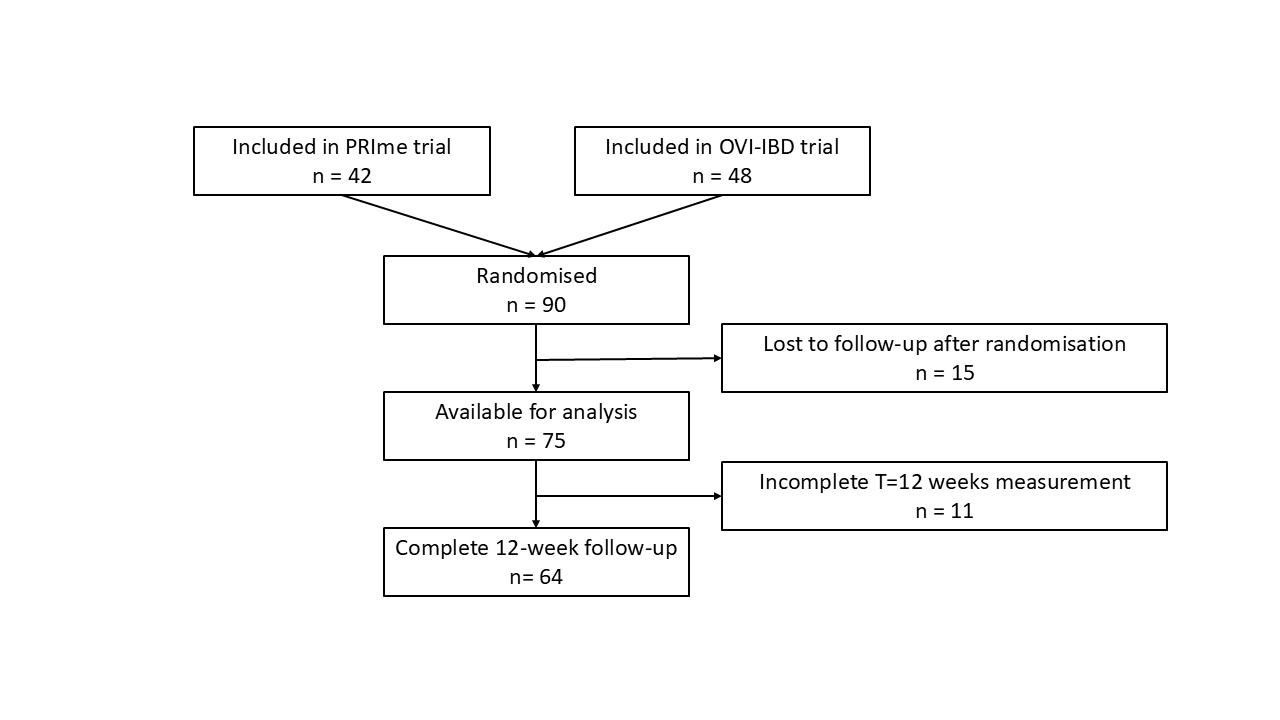


Supplementary Figure 3. Overview of the study inclusions
